# Clonality Despite Sex: The Evolution of Host-Associated Sexual Neighborhoods in the Pathogenic Fungus *Penicillium marneffei*

**DOI:** 10.1371/journal.ppat.1002851

**Published:** 2012-10-04

**Authors:** Daniel A. Henk, Revital Shahar-Golan, Khuraijam Ranjana Devi, Kylie J. Boyce, Nengyong Zhan, Natalie D. Fedorova, William C. Nierman, Po-Ren Hsueh, Kwok-Yung Yuen, Tran P. M. Sieu, Nguyen Van Kinh, Heiman Wertheim, Stephen G. Baker, Jeremy N. Day, Nongnuch Vanittanakom, Elaine M. Bignell, Alex Andrianopoulos, Matthew C. Fisher

**Affiliations:** 1 Department of Infectious Disease Epidemiology, Imperial College, Norfolk Place, London, United Kingdom; 2 Departments of Microbiology, Regional Institute of Medical Sciences, Imphal, Manipur, India; 3 Department of Genetics, University of Melbourne, Victoria, Australia; 4 The Third People's Hospital, Shenzhen City, Guangdong Province, China; 5 The J. Craig Venter Institute, Rockville, Maryland, United States of America; 6 Departments of Laboratory Medicine and Internal Medicine, National Taiwan University Hospital, National Taiwan University College of Medicine, Taipei, Taiwan, Republic of China; 7 Department of Microbiology, The University of Hong Kong, Queen Mary Hospital, Hong Kong Island, Hong Kong; 8 Hospital for Tropical Diseases, Ho Chi Minh City, Viet Nam; 9 National Hospital for Tropical Diseases, Ha Noi, Viet Nam; 10 Oxford University Clinical Research Unit, Wellcome Trust Major Overseas Program Viet Nam National Hospital for Tropical Diseases, Ha Noi, Viet Nam; 11 Oxford University Clinical Research Unit, Wellcome Trust Major Overseas Programme Viet Nam Hospital for Tropical Diseases, Ho Chi Minh City, Viet Nam; 12 Department of Microbiology, Faculty of Medicine, Chiang Mai University, Chiang Mai, Thailand; 13 Department of Microbiology, Imperial College London, London, United Kingdom; ETH Zurich, Switzerland

## Abstract

Molecular genetic approaches typically detect recombination in microbes regardless of assumed asexuality. However, genetic data have shown the AIDS-associated pathogen *Penicillium marneffei* to have extensive spatial genetic structure at local and regional scales, and although there has been some genetic evidence that a sexual cycle is possible, this haploid fungus is thought to be genetically, as well as morphologically, asexual in nature because of its highly clonal population structure. Here we use comparative genomics, experimental mixed-genotype infections, and population genetic data to elucidate the role of recombination in natural populations of *P. marneffei*. Genome wide comparisons reveal that all the genes required for meiosis are present in *P. marneffei*, mating type genes are arranged in a similar manner to that found in other heterothallic fungi, and there is evidence of a putatively meiosis-specific mutational process. Experiments suggest that recombination between isolates of compatible mating types may occur during mammal infection. Population genetic data from 34 isolates from bamboo rats in India, Thailand and Vietnam, and 273 isolates from humans in China, India, Thailand, and Vietnam show that recombination is most likely to occur across spatially and genetically limited distances in natural populations resulting in highly clonal population structure yet sexually reproducing populations. Predicted distributions of three different spatial genetic clusters within *P. marneffei* overlap with three different bamboo rat host distributions suggesting that recombination within hosts may act to maintain population barriers within *P. marneffei*.

## Author Summary

Fungal pathogen populations show patterns ranging from globally recombining to endemic and clonal. Among the most genetically and spatially restricted fungi is the highly clonal pathogen *Penicillium marneffei*, an endemic AIDS-associated pathogen in Southeast Asia. Previous studies have shown that *P. marneffei* has a pattern of extreme clonality despite the ability to disperse across wide distances and the presence of mating type genes that are required for sexual recombination. In this study we used genetic markers, comparative genomics, experimental data, and spatial models to determine the influence of sex on *P. marneffei* populations, and we found that although there was substantial evidence of sexual recombination, most of the recombination in natural populations was limited to sexual neighborhoods, amongst genetically similar and spatially close individuals. Based on the results of experiments and spatial models we found support for sex occurring in bamboo rats that are known to harbor *P. marneffei* and the pathogens sexual neighborhoods. Our study suggests that the high levels of effective clonality and endemicity found in *P. marneffei* may have more to do with specific host interactions than with an innate inability to generate population genetic diversity through sexual recombination.

## Introduction

Hypotheses of globally continuous populations and strict clonality in putatively asexual microbial pathogens are rarely supported [1], [2], [3], [4], [5]. Instead, genetic approaches detect recombination in microbes regardless of assumed asexuality, and pathogens are surprisingly promiscuous despite strong population genetic structure [6], [7], [8]. In some eukaryotic pathogens spatial structuring is readily attributable to dispersal limitations [9], [10], but many fungal pathogens of humans display extensive spatial population genetic structure despite their ability to disperse via aerosolized spores [11], [12], [13], [14], [15]. Examples that cause extensive morbidity include *Cryptococcus neoformans*, *Coccidioides* sp., *Histoplasma capsulatum* and *Penicillium marneffei*. These fungi are maintained in natural environmental reservoirs that might contribute to structured populations via local adaptation, and they are thought to be largely clonal. However, fungi have many different mating systems that encompass asexual propagation t7hrough multiple forms of sexual and parasexual recombination, and clonal structure may be arrived at via very different mechanisms [16], [17]. Evidence suggests that population structure in fungal pathogens is strongly influenced by host distributions and extrinsic geographic boundaries [12], [18], [19], [20], [21]. Therefore, the interplay between mating systems, population structure, and host adaptation is a central question underpinning the evolutionary epidemiology of fungal pathogens.

Heterothallic mating systems in fungi require physical contact between two isolates containing opposite mating types at the mating-type locus (*MAT*) in order to undergo sexual reproduction. If no mating partners are present, then sexual reproduction does not occur and the fungus reproduces asexually (but see Lin et al. [22] for evidence of same-sex mating in the otherwise heterothallic fungus *Cryptococcus neoformans*). In this case, the relative capacities of fungal lineages to disperse and co-occupy environmental niches can drive population-level recombination rates. If strains of opposite mating type do not equally penetrate environments then species recombination rates may be reduced to levels nearing complete asexuality [23]. Previously, it has been shown that the HIV-associated emerging pathogen *Penicillium marneffei* shows extensive spatial genetic structure at local and regional scales across Thailand [XPATH ERROR: unknown variable "start2".], [24]. Although there has been some genetic evidence that a sexual cycle is possible in *P. marneffei*, this haploid fungus is thought to be genetically, as well as morphologically, asexual within these populations [25].

In this study, we use comparative genomics, experimental approaches, and population genetic data to identify the role of sexual recombination in maintaining spatial and genetic structure in this infection. We attempt to answer 4 specific questions: 1) Does the *P. marneffei* genome show evidence of sex? 2) How are populations of *P. marneffei* genetically structured? 3) Can population structure be reconciled with sex? 4) Do spatial or host factors correlate with population structure and sex? We use comparative genomics to identify genes linked to mating and genomic signatures of mutation bias associated with meiosis, and we experimentally detect recombination *in vivo*. We expand our collection of population genetic data across southeast Asia to include mating type data and 34 isolates from bamboo rats in India, Thailand and Vietnam, and 273 isolates from humans in China, India, Thailand, and Vietnam. Together these data form a mosaic that reveals some physical and genetic underpinnings of mating in *P. marneffei* that are linked to patterns of genetic diversity across its known endemic range.

## Materials and Methods

### Genomic analyses

We used 84 sexual cycle genes (Table S1) to blast against the NCBI genome sequences NZ_AAHF00000000 (*A. fumigatus*), NZ_ABAR00000000 (*P. marneffei*), and NZ_ABAS00000000 (*T. stipitatus*). We screened transposon families for substitution bias by making BLAST based alignments to determine the dominant form of a functional integrase gene in each family. We counted the type of substitution based on differences of alleles as low as 70% identical to the dominant intact type. We compared gene sequences of the genomic region between *slaB* and *apnB* (the genes that flank the *MAT* idiomorph in related fungi) of strains FRR2161 and FRR3842.

### Isolates, MLMT barcoding and *MAT* discrimination

We acquired 307 isolates of *P. marneffei* from humans and vertebrate hosts (bamboo rats), covering the known global range of the fungus. Our study obtained 273 epidemiologically unlinked human isolates of *P. marneffei* from HIV-AIDS patients covering the time-period 1959 to 2005. Of these isolates, 258 were georeferenced to either the broad geographical region of collection or the patients home address. The remaining 15 isolates were recovered from patients whose infections were diagnosed in non-endemic regions, and no accurate geographical origin could be assigned. In addition to human isolates of *P. marneffei*, we obtained 34 isolates from the bamboo rats species *Rhizomys pruinosis* (*n* = 3), *R. sumatrensis* (*n* = 13), *R. sinensis* (*n* = 1) and *Cannomys badius* (*n* = 17). We also include the type isolate for *P. marneffei* Segretain et al. ATCC 18224, CBS 388.87, isolated from *R. sinensis* in 1959 [26]. All isolates were cultured on Sabouraud's agar and DNA extracted as previously described [27]. Subsequently, isolates were genotyped at 21 microsatellite loci using the methods described in Fisher *et al.* [11], [27].

The presence within each of isolate of the *MAT1-1* α box and *MAT1-2* high mobility group idiomorphs was determined using the PCR protocol detailed by Woo *et al.* [25].

### Population genetic structure and spatial analyses

Genotypes were analysed using GenAlex 6.0 [28] to determine allelic diversity, genotypic diversity and spatial correlation across regions and the global distribution of *P. marneffei*. We used the package adegenet and its dependencies in R to conduct spatial PCA and DAPC analyses [29]. To compare our inferred results against a model of a single continuous population structured by a dingle dispersal kernel and mutation rate we used the coalescent based program IBDsim [30]. Additional distribution data for bamboo rats were collected from specimen databases AMNH, FMNH, NMNH, and the GBIF. We used the bioclim layers 1–21 at 30 sec from the world clim database in MAXENT to generate predicted distributions for bamboo rats and *P. marneffei* genetic clusters. We measured distributional overlap using Schoener's *D* and a resampling approach [31]. We compared the relative overlap of genetic clusters to host distributions by generating null distributions of *D* based on resampling of *R. sumatrensis* and *Cannomys* (Text S1). Possible parental distances were compared to null distributions generated by choosing isolates randomly that met the genetic distance criteria from the population that met the parental criteria.

### Co-infection analysis

Five co-housed outbred CD-1 male mice (16–18 g) were inoculated intranasally with 10^7^ spores suspended in 40 µl of PBS. Conidia from two isolates, PM9, a *MAT* 1–2 isolate from Thailand, and the type strain ATCC18824 (FR2161) were mixed in a 1∶1 ratio to form the inoculum (S9). Serial dilutions of homogenized saline samples were plated (no later than 6 hours after they were removed from the mice) on Sabouraud agar. Colonies were counted after 4 days in 27°C. Individual colonies used for DNA extraction and subsequent genotyping as before [11]. Isolate genotypes were compared to the initial genotypes of the inoculum and genotypes differing from inoculum were confirmed via DNA sequencing.

### Ethics statement

All the clinical studies from which isolates are available were approved by the Wellcome Trust ethics committees at the study sites, in the UK and by the regulatory authorities of the countries involved. All patients or their next of kin gave written informed consent and all patient data are anonymised. This work strictly complied with the animal regulations and guidelines under UK law and was approved by Imperial College's Ethical Review Process (ERP) Committee and the British Home Office. All murine work was carried out in a Biosafety level 3 secure animal facility under licensed approval from the British Home Office.

## Results/Discussion

### Genomic evidence for sex in *P. marneffei*

**Figure 1:**
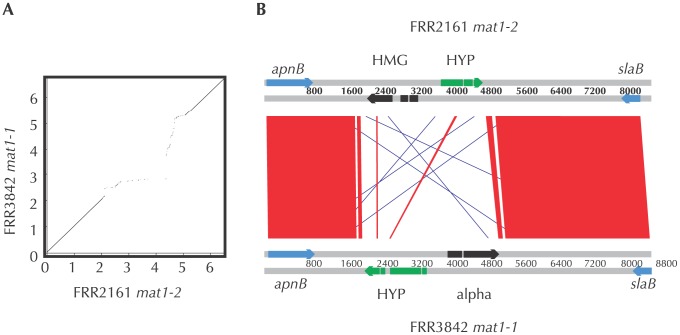
Mating type idiomorph of *P. marneffei*. A) A dot plot showing similarity between strains FRR2161 and FRR3482 similarity across the 7 kb covering MAT loci. B) Gene cartoon showing arrangement of genes in the MAT idiomorphs. Grey regions are areas of high homology, and black lines are small segments of rearrangement. Coding regions of MAT alpha and HMG genes are shown in black, flanking genes with high homology are shown in grey, and hypothetical genes with low homology are shown in white. Arrows indicate the direction of transcription.

**Figure 2:**
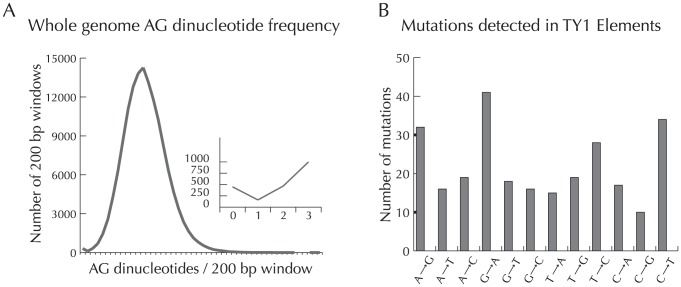
Biased mutation in *P. marneffei*. Nucleotide bias in *P. marneffei*. A) AG dinucleotide frequency across the entire genome of P. marneffei shows a non-normal distribution consistent with patterns of RIP-like process B) Comparison between the types of mutations from the inferred ancestral state shows a bias in transitions and towards G to A and C to T mutations in particular.

Sexual reproduction leaves an imprint on fungal genomes by maintaining genes required for mating and by generating patterns of mutation and recombination restricted to meiotic processes [32], [33], [34], [35], [36]. Successful mating in fungi requires that a genome contains a functioning series of interconnected genetic pathways [37]. Using a comparative genomic approach we assessed the presence and functionality in *P. marneffei* of genes known to be involved in sexual development in fungi. First, comparing between strains FRR2161 and FRR3842 revealed that the region between genes *slaB* and *apnB* resembled other fungal mating type idiomorphs. A region of complete dissimilarity was flanked by regions that were nearly identical between the strains (Fig. 1A and B). We found homologs for nearly all of the genes needed for a complete sexual cycle in yeast to be present and putatively functional (Table S1). Those genes not detected in *P. marneffei* were also not detected in *Talaromyces stipitatus*, a fungus with a complete sexual cycle, and most genes that were absent in those two fungi were also missing in the recently demonstrated heterothallic fungus *Aspergillus fumigatus*. Although these sex-related genes may be conserved to function in processes other than mating, their presence suggests that *P. marneffei* has preserved the ability to complete a sexual cycle. We detected another genomic signature of a functional sexual cycle, a type of mutation bias associated with meiosis. Repeat induced point mutation (RIP), a process by which some fungi silence genes involved in mobile genetic element function by preferentially mutating repeated sequences within their genomes, is associated with meiosis [38]. This process results in skewed base pair distributions due to the induced mutations. Using an approach similar to that of Clutterbuck [39], we found evidence of an excess of sliding windows with zero AG and CT dinucleotides and mutation bias in *P. marneffei* transposon family Ty-1 with a skew towards G to A and C to T transitions (Fig. 2). We also detected a putatively functional RID gene (Locus ID PMAA079888), the only conserved gene so far implicated in RIP [40], [41], [42]. Although the RID gene and the observed mutation bias can be explained by several factors including those acting during mitosis, they point towards a RIP or RIP-like process that is generally considered a feature of sexually reproducing fungi and an overall genomic pattern consistent with sex. Although these genomic signatures could represent relics from a sexual past rather than ongoing sexual recombination within *P. marneffei*, in the related human pathogenic fungus *Aspergillus fumigatus*, the discovery of mating type genes and evidence of RIP heralded the eventual description of a full sexual life cycle [35], [43], [44].

### Spatially correlated genetic diversity

**Figure 3:**
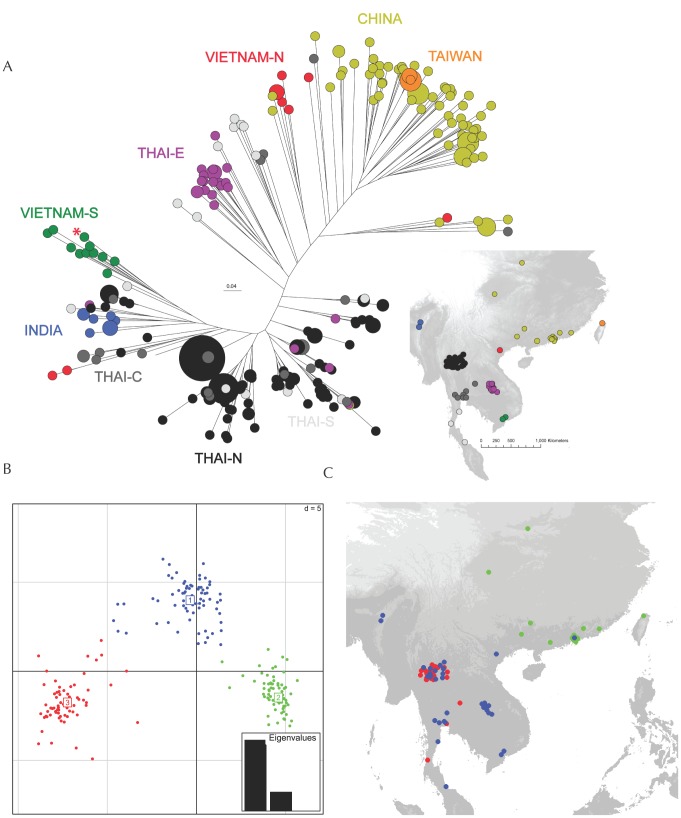
Correspondence between genetic diversity and spatial location. A) Unrooted neighborjoining tree of genetic distances between isolates labelled with the colour each isolation locality. B) Genetic clusters inferred using DAPC and isolates coloured according to cluster. C) Spatial distribution of DAPC clusters.

Microsatellite allelic diversity was high overall and within localities (Table 1). With the exception of Thai Central and Thai South, populations assigned *a priori* by locality were significantly differentiated from one another by Wright's *F_ST_* [45]; this metric ascertains the proportion of genetic variance among geographical regions relative to the total variance. *F_ST_* values near zero mean that populations are not distinct and variation is shared equally within and between them, while higher *F_ST_* values mean that more genetic differences occur between populations compared to those within populations. The China and Taiwan populations were most different from the other *a priori* populations (Table 2). Phylogenetic analysis revealed associations between sampling area and the occurrence of phylogenetic clustering (Fig. 3). Using discriminant analysis of principal components (DAPC) to identify genetic clusters [46], [47], we assigned individuals to 3 clusters based on the Bayesian information criterion. The clusters show some spatial association. Cluster 1 is composed mostly of isolates from central and southern Thailand, Cluster 2 of isolates from China, and Cluster 3 of isolates from northern Thailand (Fig. 3, Figure S1). As expected, we observed a strong pattern of spatial genetic correlation (*r* ^2^ = 0.41, p<0.01). We also detected significant ‘global’ genetic structure (positive correlation between spatial and genetic distance) but no ‘local’ genetic structure (negative correlation) using spatial principal coordinate analysis [46], [47]. To test for a homogenous neutral process of genetic differentiation we used a spatially explicit coalescent-based simulation of isolation by distance generated with IBDsim [30] to simulate a uniform dispersal/mutation process across our exact sampling scheme. This uniform genetic structure was then compare against our recovered spatial genetic pattern. By controlling for the spatial distribution of our sample sites we are able to determine if the apparent genetic clustering is simply an artifactual product of clustered sampling and a single uniform process of genetic clustering. Because the hypothesised parameter space is nearly infinite, we concentrated on dispersal scenarios that most closely resembled the spatial genetic correlation present in our data, namely, the strength of spatial genetic correlation at the smallest spatial scale and the decay rate of the correlation. Although the simulated datasets largely overlapped with our recovered data we observed important departures between the two. The simulated datasets had a single peak in spatial genetic correlation at the smallest spatial scale and a decay in correlation dependent on the dispersal kernel, a feature of all single population isolation-by-distance models, but the observed pattern had additional peaks in certain distance classes that disrupted the uniform decay (Fig S2 and S3). One peak was composed of distances between individuals belonging to the outer edges of clusters 1 and 3. Another major peak comes at the spatial scale where the outer edges of Clusters 1 and 3 contact with Cluster 2. These results differ from previous results that observed different rates of decay for spatial genetic correlation [24], a feature that probably owes to the limited geographic scope and power of the earlier study. Our data now suggest that the observed genetic clusters are not the result of a process of uniform decay with geographic distance, and that other factors are also driving the heterogeneity observed in our dataset.

**Table 1:** Local population diversity.

Population	*n*	MT	*MAT1-1*_(%)_	*MAT1-2*_(%)_	*N_a_*	*N_e_*	*H_e_*	*P_a_*
CHINA	66	57	42_(70)_	20_(30)_	5.524_(0.872)_	2.716_(0.519)_	0.519_(0.050)_	1.238_(.625)_
INDIA	11	6	7_(77)_	2_(22)_	1.857_(0.186)_	1.582_(0.282)_	0.282_(0.055)_	0.095_(0.066)_
THAI-C	22	19	16_(76)_	5_(34)_	3.952_(0.417)_	2.281_(0.470)_	0.470_(0.044)_	0.048_(0.048)_
THAI-E	25	23	4_(44)_	5_(56)_	3.619_(0.519)_	2.344_(0.434)_	0.434_(0.056)_	0.190_(0.088)_
THAI-N	120	62	72_(70)_	31_(30)_	3.619_(0.434)_	1.834_(0.331)_	0.331_(0.057)_	0.333_(0.159)_
THAI-S	19	18	9_(47)_	10_(53)_	4.095_(0.539)_	2.726_(0.515)_	0.515_(0.047)_	0.429_(0.235)_
TAIWAN	8	3	8_(100)_	0_(0)_	1.095_(0.066)_	1.042_(0.028)_	0.028_(0.020)_	0.000_(0.000)_
VIETNAM-N	10	8	4_(50)_	4_(50)_	3.333_(0.319)_	2.561_(0.512)_	0.512_(0.052)_	0.238_(0.136)_
VIETNAM -S	11	11	1_(9)_	10_(91)_	2.667_(0.374)_	2.073_(0.363)_	0.363_(0.061)_	0.048_(0.048)_
TOTAL	292	197	163_(65)_	87_(35)_	3.307_(0.177)_	2.129_(0.384)_	0.384_(0.020)_	

*n*, number of samples; *MT*, number of microsatellite types ( = haplotypes); *N_a_*, mean number of alleles; *N_e_*, mean effective number of alleles ; *H_e_*, mean haploid genetic diversity; *P_a_*, mean number of private alleles. Standard errors are shown in parentheses for *N_a_*, *N_e_*, *H_e_* and *P_a_*.

**Table 2:** Local population differentiation.

	CHINA	INDIA	THAI-C	THAI-E	THAI-N	THAI-S	TAIWAN	VIETNAM-N	VIETNAM-S
CHINA		0.815	0.716	0.706	0.927	0.752	0.277	0.412	0.808
INDIA	**0.386**		0.268	0.373	0.322	0.257	1.211	0.416	0.462
THAI-C	**0.319**	**0.220**		0.214	0.048	0.070	1.118	0.414	0.471
THAI-E	**0.332**	**0.301**	**0.154**		0.216	0.189	1.240	0.532	0.434
THAI-N	**0.462**	**0.345**	**0.059**	**0.242**		0.077	1.304	0.576	0.487
THAI-S	**0.309**	**0.196**	0.019	**0.123**	**0.097**		1.023	0.417	0.350
TAIWAN	**0.267**	**0.746**	**0.562**	**0.595**	**0.647**	**0.527**		0.740	1.214
VIETNAM-N	**0.200**	**0.283**	**0.208**	**0.276**	**0.405**	**0.183**	**0.521**		0.641
VIETNAM-S	**0.359**	**0.382**	**0.290**	**0.297**	**0.410**	**0.214**	**0.687**	**0.317**	

*F_ST_*, the proportion of variance among geographical regions relative to the total variance. Pairwise population values of *F_ST_* are below the diagonal with significant values (*p*<0.001 from 999 permutations) in bold. Pairwise Nei's genetic distances are shown above the diagonal.

### Recombination and linkage disequilibrium

Linkage disequilibrium was high throughout the sample with an overall 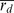 of 0.113 (Table S2). We determined the relative frequency of mutation to recombination using the single locus variant approach applied by Fisher *et al.* [24] and found a mutation to recombination frequency of 0.083 suggesting that mutation is up to 12 times less frequent than recombination across the whole population. When restricted to only bamboo rat isolates, all single locus variants would be due to recombination, while for human-only isolates the ratios are unchanged in comparison to the entire dataset. Average fungal microsatellite mutation rates have been inferred from between 2.80×10^−6^ and 2.50×10^−5^ mutations per generation [48], making the inferred recombination rate in *P. marneffei* between 3×10^−5^ and 2.50×10^−4^, a rate about half that observed in wild yeast [49]. This approach only detects single locus recombination events, which may be a minority in eukaryotic populations, while it should detect virtually all mutations that have not otherwise been masked by recombination. However, the method could be strongly biased towards inferring recombination due to convergent mutations in microsatellite length. The measure of minimal recombination (R_M_), which represents the minimum number of recombination events necessary to explain alleles failing the four gamete test [50] given the arrangement of the alleles in a contig, showed that recombination did occur within contigs (Figure S4). Complete clonality and complete panmixia are rejected for *P. marneffei*, but similar to previous results the inferred levels of clonality remain among the highest observed for fungi [24]. To explain the high level of clonal structure either recombination must be rare or it must occur largely between closely related individuals.

### Distribution of *MAT* genes

The entire sample population of *P. marneffei* showed a distribution of mating types that was significantly skewed (p = 0.02 or p = 0.04 when clone corrected) from a ratio of 1∶1 in favour of an overabundance of *MAT1-1* alleles, but some local populations were skewed towards *MAT1-2* alleles (Table 1). Two of the genetic clusters inferred by DAPC were skewed towards more *MAT1-1* alleles, but *MAT* genes within the central cluster did not differ from a 1∶1 ratio (Table 3). As predicted in work prior to the discovery of *MAT* loci in *P. marneffei*, highly skewed *MAT* ratios would be expected in a predominately asexual population [24]. On one hand, in the absence of sex and selection, *MAT* genes at an initial frequency of 0.5 are expected to be fixed in a population on average by ln 2(*N*_e_) generations. Alternatively, in a completely sexual population without selection associated with a mating type, *MAT* alleles would be maintained at frequencies near 0.5 with very limited variance because all individuals in each generation will possess *MAT* alleles according to a binomial distribution, and there is no opportunity for drift beyond a single generation. *MAT* allele counts can be used to represent the reduction in effective population size caused by drift in *MAT* ratios [51], [52], but this assumes a fully sexual population. When sex is limited, the average allele frequencies for populations that do not lose sex and become fixed remain 0.5, but the variance in *MAT* allele frequency depends on population size and the frequency of sex. Based only on the differences in *MAT* allele frequencies between clusters and an intrinsic restriction on sex, *P. marneffei* would have an intrinsic upper bound of sexual recombination frequency at less than 4.5% given a modest population size of 1000. This small level of sex could explain the highly skewed ratio of *MAT* alleles in Cluster 3 and still accommodate the 1∶1 ratio in Cluster 1 while avoiding any fixation of *MAT* alleles. However, if the intrinsic sexual recombination rate explained the distribution of *MAT* alleles it would predict equal frequencies of clone detection across populations. Instead, percent clonality tracks the *MAT* allele skew, suggesting that sexual recombination in Cluster 3 is reduced relative to Cluster 1 (Table 3). We do note, however, that *MAT* allele frequencies would not be informative about where sex occurs if unisexual mating occurs in *P. marneffei* as is known in *C. neoformans* [22].

**Table 3:** Mating type counts within clusters and the percent of isolates with MLMT identical to at least one other isolate in the sample.

Cluster	Mat1-1-1	Mat1-2-1	Ne(MAT)^a^	%MTClones^b^	%MAT1-1-1 Clones^c^	%MAT1-2-1 Clones
1	33	30	1.00	10	15	3
2	47	27	0.93	19	19	19
3	74	36	0.88	40	46	28

a Ne(MAT) is the estimated effective population size based on mating type frequency within the cluster.

b MTClones are those isolates that are identical to other isolates including the MAT.

c The percent of isolates within each mating type that are identical to another isolate in the cluster.

### Sexual neighbourhoods

**Figure 4:**
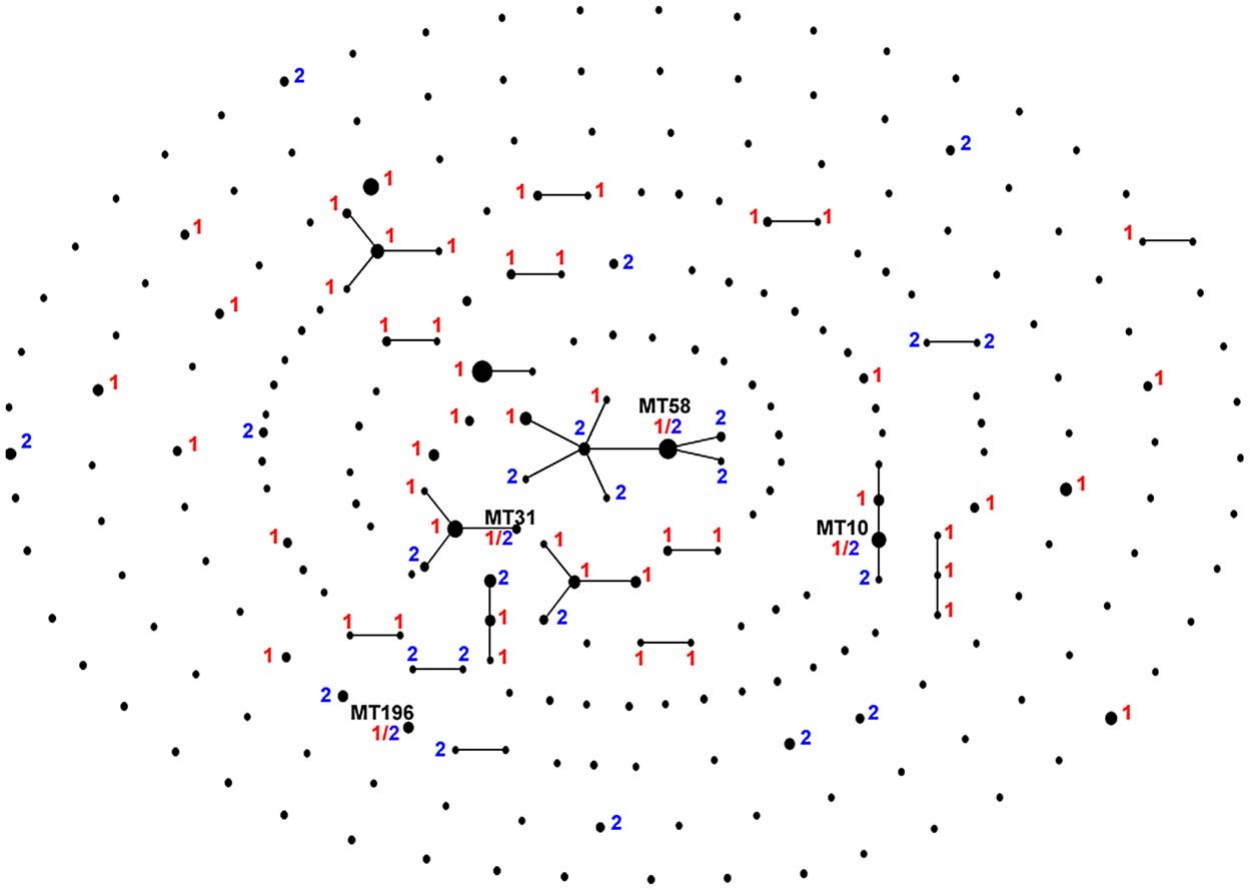
EBURST diagram showing clones with mating types. Mating types are shown for each clone composed of more than a single isolate. MAT1-1-1 isolates are labelled with a red 1, and MAT1-2-1 isolates are labelled with a blue 2. The four genotypes that have both mating types are labelled by Microsatellite Type (MT) number.

Given that recombination occurs in *P. marneffei*, we wanted to determine the geographic and genetic scope of sex. Out of 43 clonal groups inferred with EBURST [53], six contained both *MAT* alleles, and four otherwise genetically identical multi-locus microsatellite types contained both *MAT* alleles (Fig. 4). Otherwise genetically identical isolates that differ only at mating type have also been detected in *Cryptococcus gattii* populations [54], [55], [56]. Clones with both *MAT* alleles represent the smallest possible genetic scale of sex, and are unequivocal evidence for recombination. To detect the more divergent recombination events we defined putative recombinants as any genotype that had no unique alleles, yet differed from the most similar genotype for at least three loci. Putative parents or ancestral parents were defined as all isolates that together could complete the multi-locus microsatellite type (MLMT) of the recombinant genotype (Figure S5). This allows us to compare between observed distances of maximal observed recombination against a null hypothesis that any two isolates could recombine. We identified 11 potential recombinants with 54 possible parent genotype combinations. Geographic distance between putative parents was shorter, 382 km, and genetic similarity higher, 60.06% identical, than random potential parents drawn from the entire population, 675 km (p = 0.005) and 49.29% identical (p = 0.025) respectively.

**Figure 5:**
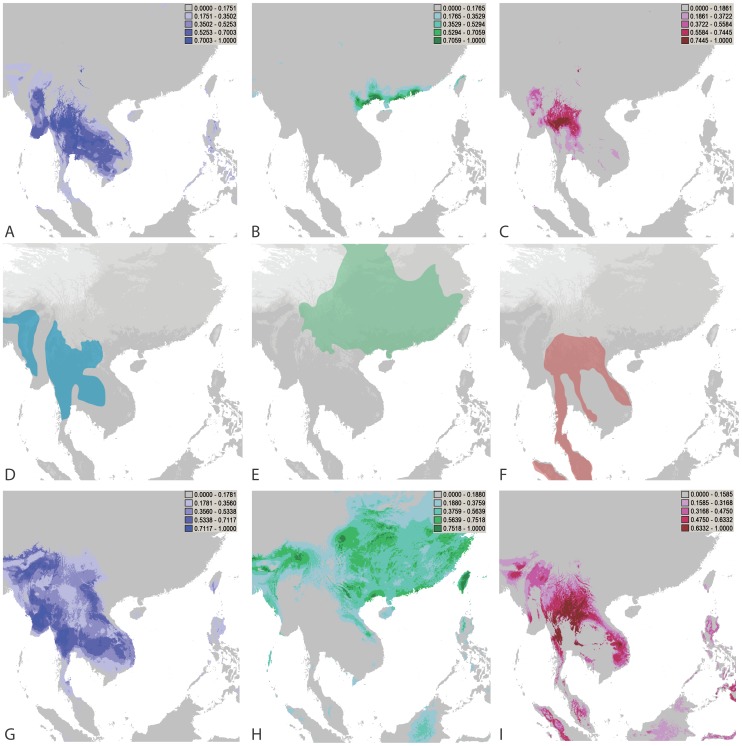
Predicted distributions of *P. marneffei* genetic clusters and bamboo rat species. A) Niche model distribution of Cluster 1. B) Niche model distribution of Cluster 2. C) Niche model distribution of Cluster 3. D) IUCN distribution of *Cannomys badius*. E) IUCN distribution of *Rhizomys sinensis*. F) IUCN distribution of *Rhizomys sumatrensis*. G) Niche model distribution of *C. badius*. H) Niche modeldistribution of *R. sinensis*. I) Predicted distribution of *R. sumatrensis*.

The scale of recombination determines the efficacy of adaptation and the adaptive potential of populations. Although recombination across large distances allows generation of greater genetic diversity and more rapid spread of advantageous alleles, it disrupts locally advantageous combinations reducing local ecological genetic correlation. When sex is limited to small geographic distances it can reinforce local adaptation, and when limited to smaller genetic distances can reinforce genomic coadaptation. Together these effects can promote ecological speciation [57], [58], [59]. When ecological adaptation acts to reinforce genetic differentiation, strong correlations between key ecological factors and population distributions will exist [60], [61], [62], [63], [64]. To assess the possibility that ecological adaptation drives population differentiation in *P. marneffei* we used MAXENT [64] to predict overlap between the ecological niches of the genetic clusters (Fig. 5). Cluster 1, the cluster with the ratio of MAT alleles nearest 1∶1, had the widest predicted range and overlapped with the entire predicted range of Cluster 3, including the predicted range that was not sampled in Myanmar. Cluster 1, Cluster 2, and Cluster 3 isolates are all found in bamboo rats, but 16 of 17 samples from *Cannomys badius* and 13 of 14 unique genotypes were from cluster 1 and distributed among India, Thai Central, Thai North, and Thai South sampling localities. None of the Cluster 1 isolates were among the 13 recovered from *Rhizomys sumatrensis*, which were all in Cluster 3. *Cannomys badius* is relatively more abundant in the western portion of the range of *P. marneffei*. The predicted distributions of bamboo rats were similar to the IUCN species ranges and had overlap with *P. marneffei* distribution. Although our spatial sample of Cluster 2 was geographically restricted it was entirely within the distribution of *Rhizomys sinensis*, a species that has been shown to consistently harbour clinically relevant *P. marneffei* [65]. The distributions for *R. sumatrensis* overlapped with *Cannomys* and Clusters 1 and 3 (Fig. 5). However, using ENMTools [66] to account for sampling error we found that Cluster 1 predicted distributions overlapped more with the *Cannomys* distribution than *R. sumatrensis* distribution, and Cluster 3 similarly overlapped more with *R. sumatrensis* than *Cannomys* distribution (S8). This observed range overlap supports a host specific effect on *P. marneffei* population structure.

Hosts may structure populations of pathogenic fungi in many ways, including by providing an environment in which recombination can occur and by acting as a selective filter on population genetic diversity [67], [68]. We used a murine inhalation model of co-infection with genetically distinct strains to investigate the effect of host infection on *P. marneffei* (Text S2). Isolates of different mating types were used for experimental co-infection of 5 mice. Subsequent culture after 15 days from the livers showed a strong bias towards recovery of the *MAT1-1* genotype for each of the mice. However, in two mice, genotypes of 4 isolates recovered from co-infections also revealed infrequent transfer of alleles between isolates of different mating type and genetic cluster (Table 4), suggesting that recombination may be possible across genetic barriers if multiple strains are within a host. In a smaller but similar *in vitro* experiment we did not observe significant bias towards *MAT1-1*, and from our scan of partial genotypes we did not recover any recombinants (S9, Table 4). We do not rule out regular recombination outside of hosts, but in the context of our spatial genetic evidence, the result of experimental infections indicate that hosts may play an important role in the development of sexual neighborhoods in populations of *P. marneffei*. However, the evolution of that role may involve restricted mating with or without host adaptation and remains to be explored.

**Table 4:** Counts of isolate genotypes recovered from murine co-infection and *in vitro* co-inoculation with ATCC18224 and PM9.

# Isolates	Organ	MAT	PM8	PM6	PM7	PM22	PM19	PM11	PM14	PM15	PM1	PM5	PM24
135	Lung/Liver	1	279	222	331	157	202	216	155	222	255	169	220
15	Lung/Liver	2	267	228	322	155	205	207	152	219	251	157	224
4	Lung	1	279	222	331	157	202	216	155	222	251	169	220
1	Lung	1+2	279	222	331	157	202	216	155	222	255	169	220
1	Lung	1+2	279	222	331	157	202	216	155	222	251	169	220
29	In Vitro	2	267	228	322	155	205	207	152	219	251	157	224
19	In Vitro	1	279	222	331	157	202	216	155	222	255	169	220
ATCC18224 (TYPE)	Bamboo Rat	2	267	228	322	155	205	207	152	219	251	157	224
PM9	Human	1	279	222	331	157	202	216	155	222	255	169	220

### Causes and consequences of sexually driven clonal structure

Asexual spores are common *in vitro* and likely a feature of natural *P. marneffei* populations, but sexual recombination may be an unexpectedly common occurrence in natural populations. The evidence supports the occurrence of recombination and perhaps even frequent sex, yet the natural populations remain strongly clonal and spatially structured. Although many mycologists might perceive this as a paradox because clonality is usually used as a proxy for asexuality, many fungi, including key pathogens, also employ same clone mating or sibling mating [7], [16], [69]. Three key hypotheses could explain the perceived clonality in *P. marneffei*; 1) Spatially restricted dispersal keeps individuals in contact with only closely related individuals; 2) Genetic incompatibility between dissimilar individuals restricts sex to genetically similar individuals; 3) Local adaptation restricts the ability of dissimilar genotypes to penetrate habitats ensuring mating between genetically similar individuals. All three are likely to be partially correct. Although the genetic evidence shows spatial limitations to effective dispersal, the physical dispersal of airborne conidia is not likely to be a limiting factor, and four genetically identical clones are dispersed across distances over 800 km. We have little information about the effect of genetic similarity on mating success in *P. marneffei*, but genetic restrictions on successful recombination are present in some plant pathogens [63], [70], [71] and should not be completely discounted. Local adaptation is not fully supported by ecological niche models that show overlap between distinct genetic clusters, but there is limited evidence of host specialisation. A key question unanswered in all of these hypotheses is why have sex at all?

Previous work has focused on the consequences of selectively neutral loss of sex in *P. marneffei* [72], but the persistence of a sexual cycle in *P. marneffei* despite abundant asexual reproduction in the lab suggests that there is a selective advantage for sex not associated with the advantages of greater adaptive potential provided by outcrossing. One major consequence of sexual clonality is release from Muller's Ratchet compared to asexuality [51]. Large numbers of haploid offspring and wide dispersal maximize environmental exposure of genets and increase the efficiency of purging deleterious alleles [73], [74]. However, the sexual process itself may also reduce the accumulation of deleterious mutations independent of recombinational effects [75]. Among the close relatives of *P. marneffei* in the subgenus *Biverticillium*, outcrossing has not been shown to occur, but self-fertility is common [76], [77], and the distribution of mating systems in the subgenus suggests that inbreeding may not reduce the evolutionary longevity of this group [78].

Another compelling scenario favouring sex recognises the opportunity presented by mating itself for dramatic shifts in morphology and physiology. A predominant view in the fungal literature is that sex occurs in otherwise mostly clonal fungi in response to stressful conditions [16], [79]. Sexually produced spores are often viable for long periods of time and are resistant to extreme environmental conditions [78], [80], [81], [82]. Regardless of the costs and benefits of recombination, *P. marneffei* might withstand stress by mating when it would otherwise not survive. If that were the case, recombination in *P. marneffei* might be clustered in space or time where or when stress occurs. Unfortunately, little is known about the natural ecology of *P. marneffei*, and any conditions that might allow mating to occur are unknown. Isolates are commonly recovered from bamboo rats, yet the epizoology of the fungus is poorly known including unknown routes of infection and unknown course and outcomes of the zoonosis. In other dimorphic fungi including *Histoplasma* and *Blastomyces*, mating in natural populations is also poorly known, but in these species it mating has long been studied outside of the host and at lower temperatures in vitro [83], [84]. Nevertheless, the association with small mammals may be the best starting point for a search for the natural sexual niche of *P. marneffei*.

Cryptic mating and inbreeding in *P. marneffei* has some parallels with other fungal and non-fungal eukaryotic pathogens [7]. There is growing support for high inbreeding in addition to asexual reproduction in *Leishmania brasiliensis* [85]. Experimental data support a role for within-vector recombination and it is thought that this restricted recombination in *Leishmania* results in sexual neighbourhoods of pathogen genotypes with high differentiation at multiple spatial scales [86]. In the malaria parasite *Plasmodium falciparum*, high inbreeding has been linked to faster emergence of drug resistance in some low infection intensity regions, but inbreeding has also emerged as a general property of *P. falciparum* populations regardless of infection intensity [87], [88]. In *Toxoplasma gondii* and *Sarcocystis neurona*, ‘clonal’ emergence is enabled by high rates of selfing and results in spatially structured populations [89], [90]. The most common fungal infection of humans, *Candida albicans*, also undergoes same-sex mating that can facilitate inbreeding and the ability to accelerate evolution via sex within clonal populations [91]. In *C. neoformans*, multiple ecological niches where recombination between strains with opposite mating type occurs have been found [92], [93]. However, most mating in *C. neoformans* globally is likely to occur between strains of the same mating type, and although there is as yet no indication of what factors alter the probability of this kind of inbreeding across natural populations, it may have facilitated the global emergence of a single mating type and highly clonal populations [15], [22], [94], [95]. Although some of the mechanisms underlying these inbreeding eukaryotic pathogens remain mysterious and likely differ between organisms, there is an emerging consistent pattern of clonality resulting from inbreeding rather than strictly asexual propagation even in the absence of a recognized sexual stage.

## Supporting Information

Figure S1 DAPC cluster analysis.
(PDF)

Figure S2 Dispersal kernel effects on spatial genetic correlation.
(PDF)

Figure S3 Simulated and observed spatial genetic correlation.
(PDF)

Figure S4 Distribution of markers and within contig recombination.
(PDF)

Figure S5 Detection of putative recombinant isolates.
(PDF)

Table S1 Mating associated genes detected in *P. marneffei*.
(PDF)

Table S2 Linkage disequilibrium.
(PDF)

Text S1 Analysis of *P. marneffei* – bamboo rat species overlap.
(PDF)

Text S2 In vitro and in vivo co-inoculation.
(PDF)
