# Supplementary material for: Clonality Despite Sex: The Evolution of Host-Associated Sexual Neighborhoods in the Pathogenic Fungus Penicillium marneffei
Source: PLoS Pathog. 2012 Oct 4;8(10):e1002851. doi: 10.1371/journal.ppat.1002851 (PMC3464222; doi:10.1371/journal.ppat.1002851)
Supplement: Figure S1 — DAPC cluster analysis. (PDF) [file ppat.1002851.s001.pdf]

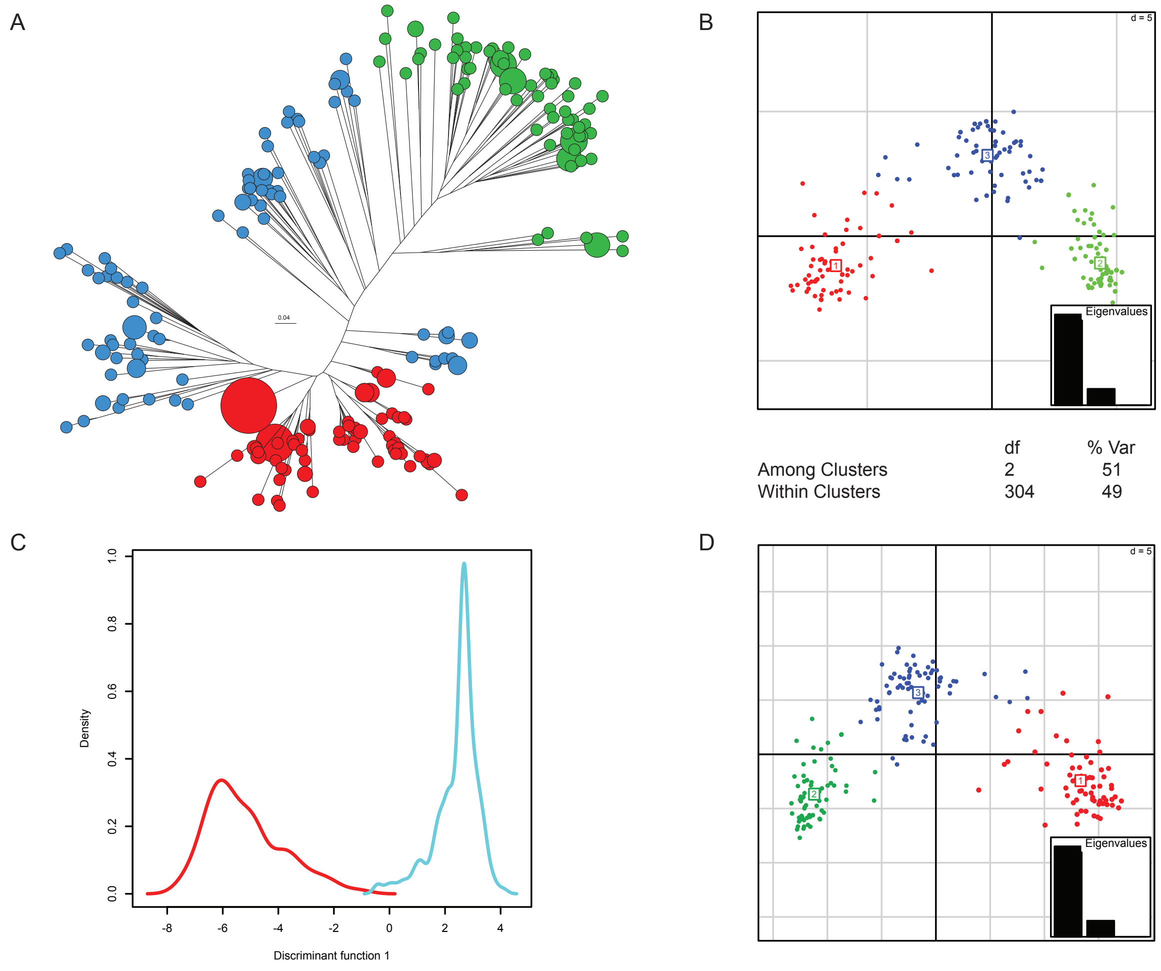

Figure S1 | DAPC cluster analysis. A) Unrooted neighbor-joining tree colored by DAPC cluster. B) DAPC clusters including only human isolates recover the same major groups, and AMOVA of these groups shows a very large portion of the variance is attributable to among cluster differences. C) DAPC analysis at only 2 clusters shows distinct but humped and stretched genetic clusters along the single axis. D) Clone-corrected DAPC analysis recovers the same clusters as uncorrected analysis suggesting that the frequency of the clones had little effect on the assignment of individual genotypes to clusters.
