# Supplementary material for: Clonality Despite Sex: The Evolution of Host-Associated Sexual Neighborhoods in the Pathogenic Fungus Penicillium marneffei
Source: PLoS Pathog. 2012 Oct 4;8(10):e1002851. doi: 10.1371/journal.ppat.1002851 (PMC3464222; doi:10.1371/journal.ppat.1002851)
Supplement: Figure S2 — Dispersal kernel effects on spatial genetic correlation. (PDF) [file ppat.1002851.s002.pdf]

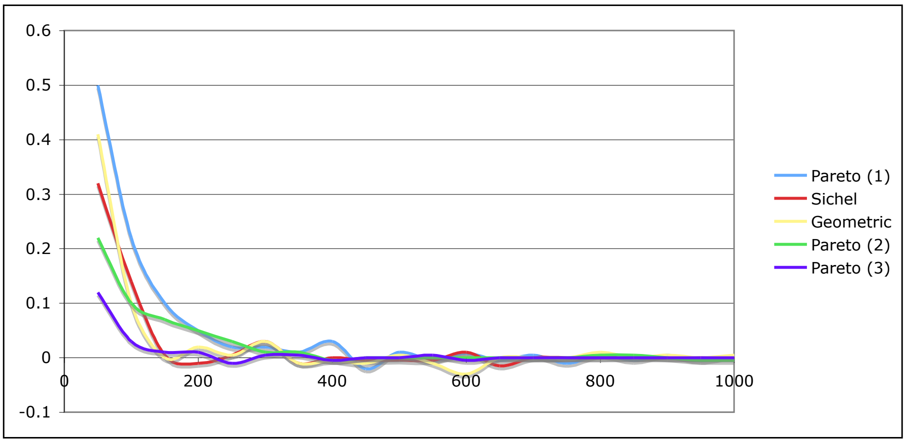

Figure S2 | Dispersal kernel effects on spatial genetic correlation. The plot of spatial-genetic correlation versus 50km distance classes shows positive r-values where the individuals in each bin that are closer in space are also more closely related. The effect of isolation-by-distance driven spatial genetic correlation decays as geographic distances become larger such that r-values approach zero when comparing between two points that are both far from the central point. Each line corresponds to the mean r-values of 10 simulated datasets for each parameter set in IBDsim [1].

1. Leblois R, Estoup A, Rousset F (2009) IBDSim: a computer program to simulate genotypic data under isolation by distance. Molecular Ecology Resources 9: 107-109.
