# Supplementary material for: Clonality Despite Sex: The Evolution of Host-Associated Sexual Neighborhoods in the Pathogenic Fungus Penicillium marneffei
Source: PLoS Pathog. 2012 Oct 4;8(10):e1002851. doi: 10.1371/journal.ppat.1002851 (PMC3464222; doi:10.1371/journal.ppat.1002851)
Supplement: Figure S3 — Simulated and observed spatial genetic correlation. (PDF) [file ppat.1002851.s003.pdf]

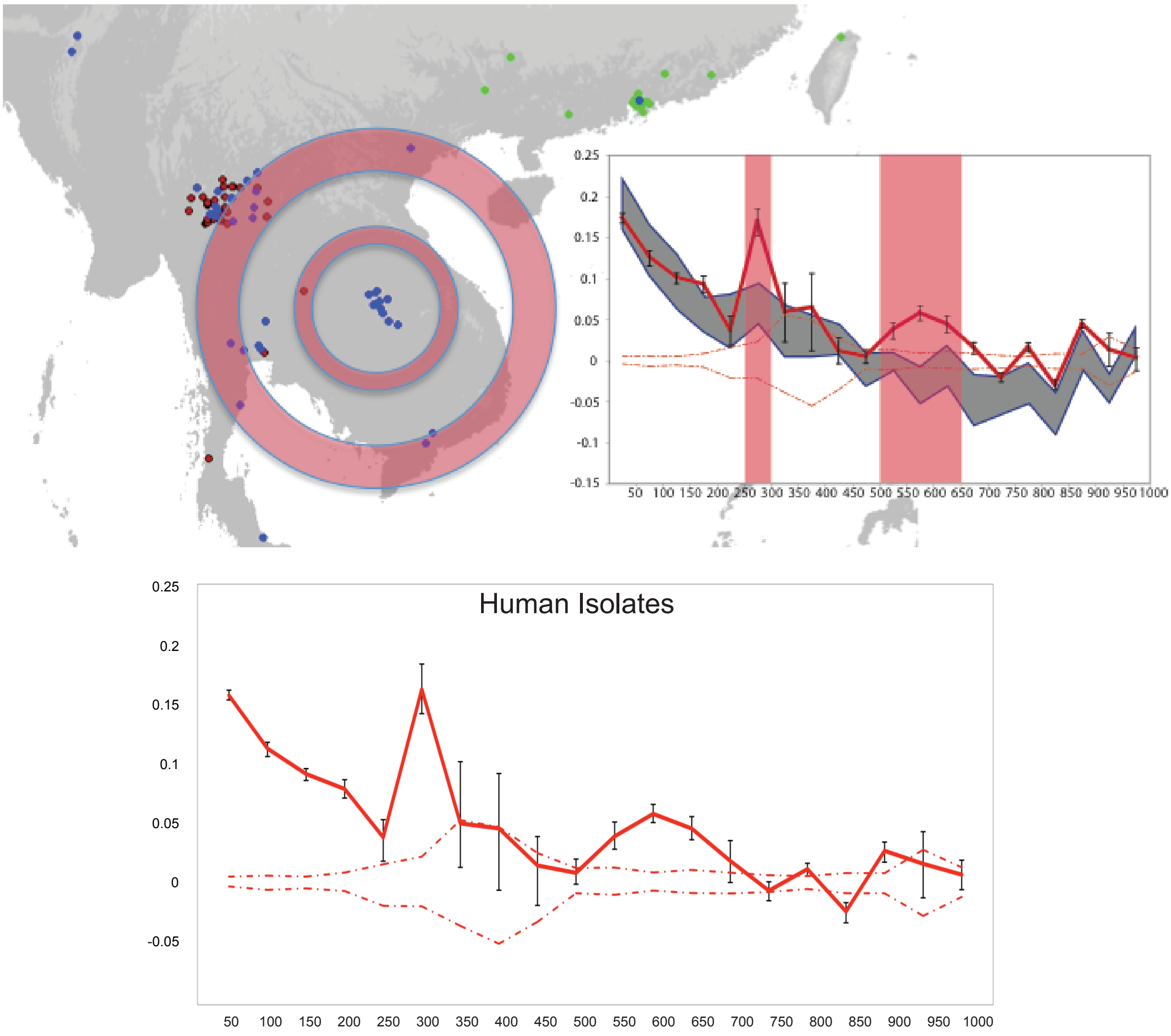

Figure S3 | Departure from single population decay in spatial-genetic correlation. The plot of spatial-genetic correlation versus 50km distance classes shows positive r-values, where the individuals in each bin that are closer in space are also more closely related. The effect of isolation-by-distance driven spatial genetic correlation decays as geographic distances become larger such that r-values approach zero when comparing between two points that are both far from the central point. In this plot, the red line shows r values from *P. marneffei*, the grey area shows the central 95% of simulated r-values from IBDsim, and the dotted red lines show bootstrap confidence intervals for no spatial genetic correlation. The map highlights areas corresponding to distance classes with departures from the uniform decay centered on a single point in Cluster 1. To generate strong correlation, points closer to the inside of a ring must be more closely related to the central point than points near the outside of the ring. To generate

the plot, the bull's-eye would need to shift across each point in the sample. The same pattern of spatial-genetic correlation and decay remains even when Bamboo Rat isolates are removed.
