# Supplementary material for: Clonality Despite Sex: The Evolution of Host-Associated Sexual Neighborhoods in the Pathogenic Fungus Penicillium marneffei
Source: PLoS Pathog. 2012 Oct 4;8(10):e1002851. doi: 10.1371/journal.ppat.1002851 (PMC3464222; doi:10.1371/journal.ppat.1002851)
Supplement: Figure S4 — Distribution of markers and within contig recombination. (PDF) [file ppat.1002851.s004.pdf]

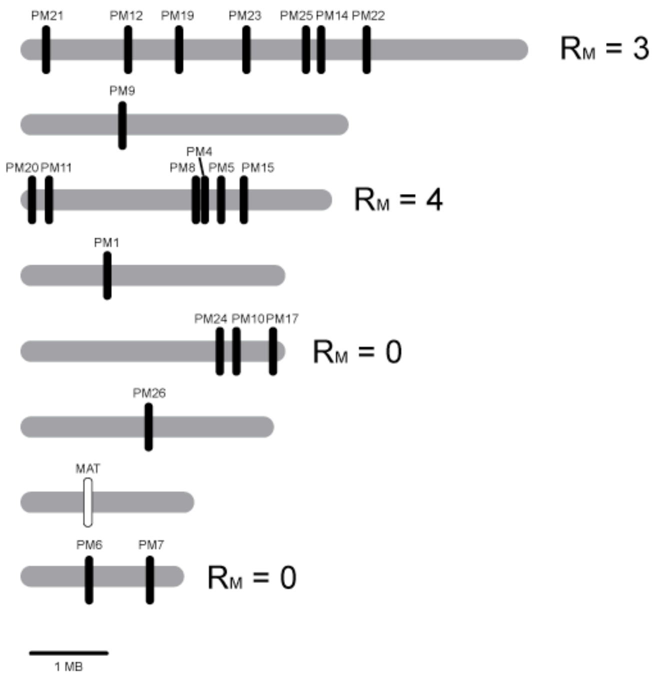

Figure S4 | Distribution of markers and within contig recombination. Each microsatellite marker is shown as a black bar in its position on one of the 8 largest supercontigs from the *P. marneffei* genome. The MAT idiomorph locus is shown as a white bar. R_M for each contig with multiple markers was determined using dnaSP [1]. For each isolate, alleles were arrayed in their order on the contig, gaps were recoded as characters and flanking sequence variation was not used because only a few isolates were sequenced for most loci, therefore RM reflects only microsatellite repeat variation.

1. Rozas J, Sanchez-DelBarrio JC, Messeguer X, Rozas R (2003) DnaSP, DNA polymorphism analyses by the coalescent and other methods. Bioinformatics 19: 2496-2497.
