# Supplementary material for: Clonality Despite Sex: The Evolution of Host-Associated Sexual Neighborhoods in the Pathogenic Fungus Penicillium marneffei
Source: PLoS Pathog. 2012 Oct 4;8(10):e1002851. doi: 10.1371/journal.ppat.1002851 (PMC3464222; doi:10.1371/journal.ppat.1002851)
Supplement: Figure S5 — Detection of putative recombinant isolates. (PDF) [file ppat.1002851.s005.pdf]

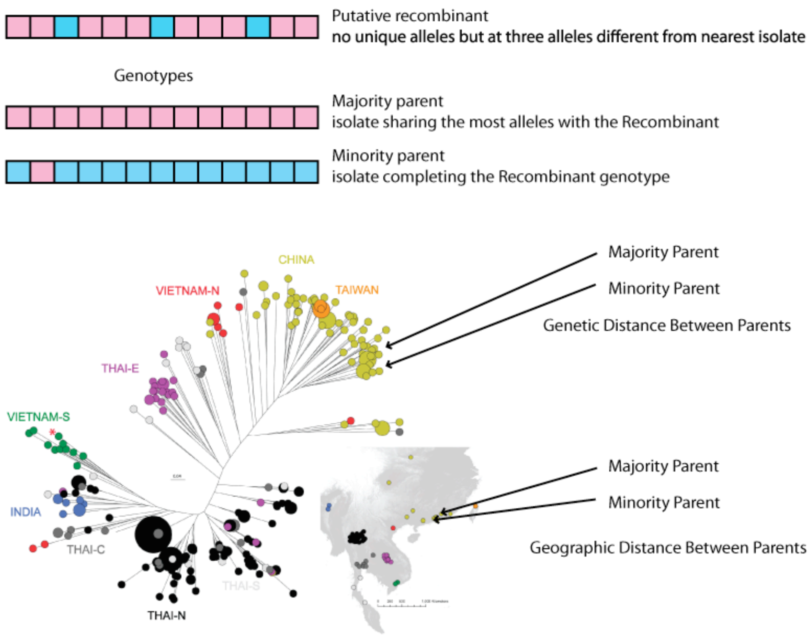


**Figure S5 | Detection of putative recombinant isolates.** By selecting a minimum of three markers, we maximized the genetic distance between recombinants, while decreasing the likelihood of convergent mutations giving a false signal of recombination. Recombinants were defined as genotypes with no unique alleles yet with three or more alleles different from the most similar genotype and sharing the alleles different from the most similar genotype with another genotype. To account for this level of both allele sharing and allelic difference requires either at least three separate homoplasy events despite identity at all other loci with its most similar genotype. To estimate the probability of this occurring in a completely non-recombining population we used Mesquite (Maddison, W.P. & D.R. Maddison. 2009 http://mesquiteproject.org) to simulate mutations across 21 loci over 100 coalescent trees from a sample of 307 isolates drawn from an effective population of $1 \times 10^5$ then applied our method to determine the frequency of false detection. No putative recombinants were detected when mutation rates were $2.50 \times 10^{-5}$ or $2.50 \times 10^{-6}$ mutations per generation. After we identified putative 'parent genotypes' as those that could combine to form the recombinant genotype. We generated a null distribution of non-parental distances by randomly choosing isolate pairs that met the genetic distance criteria imposed on the ‘parent genotypes’ from the whole population

and used KS-tests to compare the distributions of genetic and geographic distance between ‘parent genotypes’ to the null distribution of non-parental genotypes.
