# Supplementary material for: Clonality Despite Sex: The Evolution of Host-Associated Sexual Neighborhoods in the Pathogenic Fungus Penicillium marneffei
Source: PLoS Pathog. 2012 Oct 4;8(10):e1002851. doi: 10.1371/journal.ppat.1002851 (PMC3464222; doi:10.1371/journal.ppat.1002851)
Supplement: Table S1 — Mating associated genes detected in P. marneffei. (PDF) [file ppat.1002851.s006.pdf]

Table S1 | Detection of fungal mating genes in *P. marneffei*, *Aspergillus fumigatus* and *Talaromyces stipitatus* via BLAST

Meiosis		Pheromone Maturation		Phermone Response		Sexual Development	
Gene	Species	Gene	Species	Gene	Species	Gene	Species
Dmc1	**Pm**,**Ts**,Af	Kex1p/KexA	**Pm**,**Ts**,Af	Ste18p/GpgA	**Pm**,**Ts**,Af	Asd1/RhgB	Af
Hop1p	Af	Kex2p/KexB	**Pm**,**Ts**,Af	Bem1p	**Pm**,**Ts**,Af	Asd4/AreB	**Pm**,**Ts**,Af
Hop2p	None	Ram1p	**Pm**,**Ts**,Af	Cdc24p	**Pm**,**Ts**,Af	Dop1p/DopA	**Pm**,**Ts**,Af
Mlh1p	**Pm**,**Ts**,Af	Ram2p	**Pm**,**Ts**,Af	Cdc42/ModA	**Pm**,**Ts**,Af	EsdC	**Pm**,**Ts**,Af
Mlh2p	**Pm**,**Ts**,Af	Rce1p	**Pm**,**Ts**,Af	Cpc2/CpcB	**Pm**,**Ts**,Af	FlbC/Fle1	**Pm**,**Ts**,Af
Mlh3p	**Pm**,**Ts**,Af	Ste13p (DapB)	**Pm**,**Ts**,Af	CsnD	**Pm**,**Ts**,Af	FphA/Phy1	**Pm**,**Ts**,Af
Mnd1p	**Pm**,**Ts**,Af	Ste14p	**Pm**,**Ts**,Af	Dig1p/Rst1	**Pm**,**Ts**,Af	MedA/Medusa	**Pm**,**Ts**,Af
Mre11p	**Pm**,**Ts**,Af	Ste23p	**Pm**,**Ts**,Af	Dig2p/Rst2	None	Msa2p/Nrd1p	**Pm**,**Ts**,Af
Msh2p	**Pm**,**Ts**,Af	Ste24p	**Pm**,**Ts**,Af	Far1p	**Pm**,**Ts**,Af	MutA	**Pm**,**Ts**,Af
Msh4p	**Pm**,**Ts**,Af	Ste6p	**Pm**,**Ts**,Af	FMR1	**Pm***,**Ts**,Af*	NosA	**Pm**,**Ts**,Af
Msh5p	**Pm**,**Ts**,Af			Fus3p (MpkB)	**Pm**,**Ts**,Af	NsdD/Gat2p	**Pm**,**Ts**,Af
Msh6p	**Pm**,**Ts**,Af			GpaA/FadA	**Pm**,**Ts**,Af	PpoA	**Pm**,**Ts**,Af
Pms1p	**Pm**,**Ts**,Af			Gpa2p/GpaB	**Pm**,**Ts**,Af	PpoC	**Pm**,**Ts**,Af
Rad50p	**Pm**,**Ts**,Af			Ham2p/Far11/Pro22	**Pm**,**Ts**,Af	Pro1	**Pm**,**Ts**,Af
Rad51p (UvsC)	**Pm**,**Ts**,Af			Hog1/SakA	**Pm**,**Ts**,Af	Pro11/Fsr1	**Pm**,**Ts**,Af
Rad52p/RadC	**Pm**,**Ts**,Af			Kss1p (MpkB)	**Pm**,**Ts**,Af	RosA	**Pm**,**Ts**,Af
Spo11p	**Pm**,**Ts**,Af			Mat1	**Pm***,**Ts**,Af*	Rst2p	**Pm**,**Ts**,Af
				Mat2	**Pm**,**Ts**,Af	She4p/Cro1	**Pm**,**Ts**,Af
				Mlp2	**Pm**,**Ts**,Af	StuA	**Pm**,**Ts**,Af
				PpgA	Af	Swi10/Rad10	**Pm**,**Ts**,Af
				PreA (Ste3)	**Pm**,**Ts**,Af	Syg1	**Pm**,**Ts**,Af
				Prm1p	**Pm**,**Ts**,Af	VeA	**Pm**,**Ts**,Af
				RgsD	**Pm**,**Ts**,Af		
				Rri1/CsnE	**Pm**,**Ts**,Af		
				SfaD/Ste4	**Pm**,**Ts**,Af		
				SMR1	None		
				SMR2	**Pm**,**Ts**,Af		
				Sst2p/FlbA	**Pm**,**Ts**,Af		
				Ste11p	**Pm**,**Ts**,Af		
				Ste12p/SteA	**Pm**,**Ts**,Af		
				Ste2/PreB	**Pm**,**Ts**,Af		
				Ste20p/PakA	**Pm**,**Ts**,Af		
				Ste50p	**Pm**,**Ts**,Af		
				Ste5p	**Pm**,**Ts**,Af		
				Ste7p	**Pm**,**Ts**,Af		
				Ste11p/SteC	**Pm**,**Ts**,Af		

*Mat1-1 associated genes are found in separate strains in *Aspergillus fumigatus* and *P. marneffei*, while other genes are found in genome sequenced strains.

Table S1| All the known genes required for fungal mating were detected in *P. marneffei* via BLAST. Loci were considered detected when they had Expect values less than 10^{-5}. Average identity between *P. marneffei* and *T. stipitatus* mating associated genes was 79%, consistent with the genome average. *T. stipitatus* mating genes are present, and *T. stipitaus* undergoes apparent sexual reproduction, but the function of these genes has not been verified. Phylogenetic analysis has shown that this species like several other homothallic *Talaromyces* species have both Mat genes, but some other closely related species do not appear to have both genes.
