# Supplementary material for: Clonality Despite Sex: The Evolution of Host-Associated Sexual Neighborhoods in the Pathogenic Fungus Penicillium marneffei
Source: PLoS Pathog. 2012 Oct 4;8(10):e1002851. doi: 10.1371/journal.ppat.1002851 (PMC3464222; doi:10.1371/journal.ppat.1002851)
Supplement: Table S2 — Linkage disequilibrium. (PDF) [file ppat.1002851.s007.pdf]

Table S2 Linkage disequilibrium among populations of *P. marneffei*

Population	$\overline{r_d}$ (Clone corrected)	Significance (Clone corrected)
CHINA	0.046 (0.037)	< 0.001 (< 0.001)
INDIA	0.195 (0.038)	< 0.001 (0.135)
THAI-C	0.144 (0.118)	< 0.001 (< 0.001)
THAI-E	0.063 (0.052)	< 0.001 (< 0.001)
THAI-N	0.084 (0.058)	< 0.001 (< 0.001)
THAI-S	0.072 (0.065)	< 0.001 (< 0.001)
TAIWAN[a]	na (na)	na (na)
VIETNAM-N	0.205 (0.086)	< 0.001 (< 0.001)
VIETNAM -S	-0.019 (-0.019)	0.936 (0.945)
TOTAL	0.113 (0.156)	< 0.001 (< 0.001)

[a]Calculation not applicable (na) in Taiwan due to low sample-sizes.

S5|Tests for multilocus linkage disequilibrium based on nucleotide polymorphism at 21 microsatellites within the nine geographical populations of *Penicillium marneffei*.
