# Supplementary material for: Clonality Despite Sex: The Evolution of Host-Associated Sexual Neighborhoods in the Pathogenic Fungus Penicillium marneffei
Source: PLoS Pathog. 2012 Oct 4;8(10):e1002851. doi: 10.1371/journal.ppat.1002851 (PMC3464222; doi:10.1371/journal.ppat.1002851)
Supplement: Text S1 — Analysis of P. marneffei – bamboo rat species overlap. (PDF) [file ppat.1002851.s008.pdf]

Text S1 | Analysis of *P. marneffei* – bamboo rat species overlap. We wanted to determine if *P. marneffei* Clusters 1 and 3 overlapped more with one bamboo rat species than another, or if bamboo rat species did not influence the *P. marneffei* genetic cluster spatial distribution. Borrowing heavily from ideas presented in Warren et al. [1], and implemented by Jakob et al. [2] and Rödder and Lötters [3], we devised a test of alternative hypothesis based on resampling the distributional data and measuring the overlap between fungal and host distributions. We used the package ENMTools [4] to test hypotheses. We modified the 'identity test' to generate predicted distributions for each bamboo rat species and each genetic cluster under the assumption that their distributions each pair of genetic cluster and host species were identical in distribution but maintained their original sample size. This allows us to estimate the effect of sampling error in the generation of predicted distributions on the apparent overlap between genetic clusters and host species. We subtract the observed overlap (Schoener's *D*) between genetic cluster and spatial distributions from each of the simulated identity distributions to generate a distribution of relative distance scores for each hypothesis. By comparing the relative niche overlap scores we can ask 'does Cluster 1 overlap more with *C. badius* than *R, puinosus*?' and 'does Cluster 3 overlap more with *R, puinosus*than *C. badius*?' When the relative overlap distance is low then the overlap between genetic cluster and rat species is high (i.e. if one genetic cluster was from an identical distribution as a bamboo rat species, then the relative distance would be near zero) Significance is determined using a Mann-Whintey U-test. We found that Cluster 1 overlapped significantly more with *Cannomys badius* ($p \leq 0.02$) and Cluster 3 overlapped significantly more with *Rhizomys pruinosus* ($p \leq 0.05$).

1. Warren DL, Glor RE, Turelli M (2008) Environmental Niche Equivalency Versus Conservatism: Quantitative Approaches to Niche Evolution. Evolution 62: 2868-2883.
2. Jakob SS, Heibl C, Rodder D, Blattner FR (2010) Population demography influences climatic niche evolution: evidence from diploid American Hordeum species (Poaceae). Molecular Ecology 19: 1423-1438.
3. Rodder D, Lotters S (2009) Niche shift versus niche conservatism? Climatic characteristics of the native and invasive ranges of the Mediterranean house gecko (Hemidactylus turcicus). Global Ecology and Biogeography 18: 674-687.
4. Warren DL, Glor RE, Turelli M (2010) ENMTools: a toolbox for comparative studies of environmental niche models. Ecography 33: 607-611.
