# Supplementary material for: Clonality Despite Sex: The Evolution of Host-Associated Sexual Neighborhoods in the Pathogenic Fungus Penicillium marneffei
Source: PLoS Pathog. 2012 Oct 4;8(10):e1002851. doi: 10.1371/journal.ppat.1002851 (PMC3464222; doi:10.1371/journal.ppat.1002851)
Supplement: Text S2 — In vitro and in vivo co-inoculation. (PDF) [file ppat.1002851.s009.pdf]

Text S2 | In vitro and in vivo co-inoculation: Five co-housed outbred CD-1 male mice (16 – 18g) were inoculated intranasally with 10^7 spores suspended in 40μl of PBS. Conidia from two isolates, PM9, a *MAT* 1-2 isolate from Thailand, and the type strain ATCC18824 (FR2161) were mixed in a 1:1 ratio to form the inoculum. Mice received cyclophosphamide (150 mg/kg, ENDOXANA, Astra Medica) *via* intraperitoneal injection on days -3 and -1 prior to infection. On day -1, a single dose of hydrocortisone acetate (112.5 mg/kg, HYDROCORTISTAB, Sovereign Medical) was administered subcutaneously. Mice were given free access to food and water, and received continuous oral dosages of prophylactic Baytril 2.5% (Bayer) via water. Spore viability was confirmed via serial plating of the inoculating dose on Sabouraud agar composed of: 40g/l dextrose, 10g/l peptone and 20g/l agar. Mice were culled 15 days post infection, and lungs and livers were collected for detection, quantification and identification of fungal infection. 10 μl of a 1:1 mix of the strains at a concentration of 10^4 was used to inoculate 2ml of YPG broth (5g/l yeast extract, 5g/l peptone and 20g/l glucose) and grown for 2 days. Lung and liver sections were weighed and homogenized (homogenizer X120, Bennett Scientific Limited) in saline for colony counting. *In vitro* cultures were transferred to 40 ml of PBS and similarly subjected to homogenization. All murine work was carried out in a Biosafety level 3 secure animal facility under licensed approval from the British Home Office. Serial dilutions of homogenized saline samples were plated (no later than 6 hours after they were removed from the mice or cultures) on Sabouraud agar. Colonies were counted after 4 days in 27°C. Individual colonies were picked out with a probe and transferred to YPG broth and grown for 7 days at 27°C before being used for DNA extraction and subsequent genotyping as before. Isolate genotypes were compared to the initial genotypes of the inoculum and genotypes differing from inoculum were confirmed via DNA sequencing.
